# The Role of Functional Electrical Stimulation in Stroke Rehabilitation: A Preliminary Study

**DOI:** 10.1002/pri.70103

**Published:** 2025-09-01

**Authors:** Diego Piatti, Giovanni Morone, Antonio Mercuro, Stefano Paolucci, Marco Tramontano, Maria Grazia Grasso

**Affiliations:** ^1^ Santa Lucia Foundation Scientific Institute for Research and Health Care Rome Italy; ^2^ Department of Life, Health and Environmental Sciences University of L'Aquila L'Aquila Italy; ^3^ Department of Biomedical and Neuromotor Science University of Bologna Bologna Italy; ^4^ Unit of Occupational Medicine IRCCS Azienda Ospedaliero‐Universitaria di Bologna Bologna Italy

**Keywords:** balance, functional electrical stimulation (FES), gait, neuromotor recovery, rehabilitation, stroke

## Abstract

**Background and Purpose:**

Stroke often results in gait impairments such as foot drop. Functional Electrical Stimulation (FES) could promote neuromuscular recovery. This study aimed to evaluate the effects of FES integrated into conventional neurorehabilitation on gait and activities of daily living in stroke patients with foot drop.

**Methods:**

Twenty‐eight patients with stroke were enrolled and randomly allocated to either an experimental group receiving gait training with FES or a control group undergoing conventional rehabilitation. Both groups received 12 training sessions 3/week per 4/weeks. Primary and secondary outcomes were assessed at T0 and T1 using the Rivermead Mobility Index (RMI), Modified Barthel Index (MBI), Two‐Minute Walk Test (2MWT), and Timed 25‐Foot Walk Test (T25).

**Results:**

Significant improvements were observed in both groups across all outcome measures after the intervention. However, statistical comparisons between the FES and conventional therapy groups did not yield significant differences.

**Discussion:**

The addition of FES to conventional rehabilitation is an effective and safe approach stroke patients' rehabilitation.

## 1 Introduction

Stroke is a leading cause of disability worldwide, exerting a significant burden on individuals and healthcare systems (Katan and Luft 2018). Motor impairments consequent to stroke are a major determinant of diminished performance in activities of daily living (ADLs) (Kim 2022). These impairments typically manifest as muscle weakness, compromised physical coordination, and impaired balance, collectively undermining mobility and functional independence (Park and Han 2025). Among the various gait impairments observed in stroke survivors, one of the most prevalent features is foot drop, a distressing condition that predisposes individuals to falls and injuries (Stewart 2008; Morone et al. 2020). Technically, foot drop refers to the inability to achieve adequate dorsiflexion during the swing phase of gait in the hemiparetic limb, often resulting from weakness or paralysis of the dorsiflexor muscles, and is frequently accompanied by increased muscle tone of the ankle plantar flexors. Orthotic interventions are commonly employed as compensatory measures in the management of foot drop, providing mechanical support to improve gait stability (Yamaguchi et al. 2024). Concurrently, physical therapy remains a cornerstone of stroke rehabilitation, enhancing neuromuscular control through the targeted strengthening of the paretic muscles, improved postural stability, and refined motor coordination, thereby facilitating the functional reorganization of affected motor networks (Kang et al. 2021). Rather than operating as isolated or conflicting approaches, orthotic interventions are integrated within this rehabilitative framework, providing mechanical support and alignment that synergistically complement physical therapy to optimize overall functional recovery. In the context of synergistic approaches, it is important to consider an additional paradigm for ameliorating foot drop in stroke patients: functional electrical stimulation (FES). FES involves applying electrical impulses to the affected muscles, thereby inducing coordinated contractions that facilitate the execution of functional movements or tasks (Atkins and Bickel 2021). Moreover, FES can be categorized into three primary classes based on its intended purpose: restoration of sensory functions, restoration of motor functions, and restoration of autonomic functions, for example, for autonomic dysreflexia in patients with high spinal cord lesions (Rushton 1997; Zhou et al. 2025). Previous reviews have underlined the potential of FES in post‐stroke rehabilitation (Kang et al. 2021; Zhou et al. 2025); however, they call for further studies to identify optimal treatment protocols, stimulation parameters, and moderator variables to enhance clinical efficacy (Zhou et al. 2025). In light of this, our hypothesis is that incorporating electrical stimulation into routine rehabilitation protocols can improve motor abilities in stroke patients. For these reasons, this study aims to evaluate the effect of FES on global mobility, gait velocity and capacity and enhance activities of daily living (ADLs) in stroke survivors.

## 2 Methods

### 2.1 Study Design

This study is a two‐arm single‐blind randomized controlled trial.

The research was conducted at Santa Lucia Foundation (Institute for Research and Healthcare) from September 2016 to December 2024. The study was conducted following the CONSORT guidelines for good clinical practice (Schulz et al. 2010) and was approved by the independent Ethics Committee of Santa Lucia Foundation with the protocol number Prot. CE/PROG.519 28‐09‐15. All patients provided written informed consent to participate.

Participants were randomly assigned to the experimental group (FESg) and the control group (CTRg).

### 2.2 Participants

Twenty‐eight stroke patients were enrolled through the neurorehabilitation unit of the Santa Lucia Foundation and included in the study. The inclusion criteria comprised patients presenting with foot drop due to the stroke aged between 25 and 80 years. Additionally, all patients underwent a clinical assessment by a neurologist specialized in rehabilitation to identify and exclude potential confounding factors that could interfere with the study.

Exclusion criteria included participation in other research studies, severe comorbidities such as diabetes, vestibular disorders potentially affecting balance, and underlying neurological conditions such as multiple sclerosis or Parkinson Disease, presence of a cardiac pacemaker, pregnancy, and significant spasticity of the ankle that limited passive mobilization (Modified Ashworth Scale score > 3). Additionally, patients with ankle arthropathy restricting dorsiflexion beyond 0°, or with severe muscle weakness of the ankle and foot (MRC ≤ 1), were excluded. Muscle strength was assessed clinically by both a physiotherapist and a neurologist. Patients with skin lesions at the FES application site were also excluded. Patients with skin lesions at the FES application site were also excluded from the study.

Randomization was performed using a computer‐generated sequence to ensure unbiased allocation. This process was carried out by an independent researcher who was not involved in the intervention sessions. The randomization was done to minimize selection bias and ensure that both groups were comparable at base‐line.

### 2.3 Interventions

The experimental protocol ensured that both the FESg and CTRg received the same total duration of neuromotor rehabilitation throughout the study period. Specifically, both groups participated in an additional 12 treatment sessions (three sessions per week for four weeks), each lasting 40 min, in conjunction with their standard rehabilitation program at their neurorehabilitation units.

The CTRg underwent 12 sessions of conventional gait training, while the FESg received 12 sessions of gait training incorporating a Foot Drop Stimulator (FDS).

All patients were hospitalized or enrolled in a Day Hospital program and followed an individualized rehabilitation plan tailored to their specific impairments and residual functional abilities as part of their standard care.

Experimental treatments consisted of gait training with the application of the Bioness L300 FDS (Bioness L300; Bioness Inc.). The Bioness L300 is an advanced FES system that consists of a wireless, ergonomically designed lower leg cuff positioned just below the knee, containing electrodes that provide targeted stimulation at 20–50 Hz frequency. Additionally, a gait sensor detects walking patterns and adjusts stimulation parameters in real time to accommodate varying speeds and surfaces. After allowing the patient to wear the device, the stimulation settings were adjusted based on patient feedback and visual feedback from the therapist, ensuring optimal dorsiflexion and eversion responses. Patients then performed 40 min gait training sessions with FES.

Conventional therapy consisted of standardized neuromotor rehabilitation sessions designed to promote muscle recruitment and improve gait patterns through therapist‐guided exercises. Following these targeted interventions, patients engaged in supervised gait training, where step patterns were monitored and corrected as needed by the therapist to optimize functional mobility.

### 2.4 Outcome

At enrollment, clinical and demographic data were collected. A blinded examiner assessed both primary and secondary outcomes. All patients underwent evaluations at baseline (T0) and after completing 12 training sessions (T1). The primary outcome was the change in functional mobility, assessed using the Rivermead Mobility Index (RMI), a validated scale for evaluating global mobility in neurological patients, with scores ranging from 0 (poor mobility) to 15 (good mobility) (Lee et al. 2020). Secondary outcome measures included: Modified Barthel Index (MBI): assessing independence in activities of daily living, with scores ranging from 0 to 100 (Castiglia et al. 2017); Two‐Minute Walk Test (2MWT): measuring walking endurance based on the total distance covered within two minutes (Selvam et al. 2024); Timed 25‐Foot Walk Test (T25): assessing gait speed over a standardized distance of 7.62 m (25 feet) (Motl et al. 2017). All clinical assessments were performed by an independent investigator, blinded to group allocation and not involved in the intervention sessions.

### 2.5 Statistical Analysis

A statistical analysis was conducted to evaluate the effects of the intervention on the primary and secondary outcome measures. The normality of the data was first assessed using the Shapiro‐Wilk test to determine whether parametric or non‐parametric tests should be applied. The Wilcoxon signed‐rank test was employed to assess the within‐group changes from baseline (T0) to post‐intervention (T1) for each group. This test was applied to the data for both the FES and CTR groups, comparing pre‐intervention and post‐intervention values. The Wilcoxon test was used to evaluate whether there were significant changes in the outcome measures within each group.

To compare differences between the two groups (FES vs. CTR) at both time points (T0 vs. T1), the Mann‐Whitney *U* test was applied. This test was used to assess if there were significant differences in the outcome measures between the FES and CTR groups at baseline (T0) and after the intervention (T1). All statistical analyses were performed using the Python programming language, and significance was set at *p* < 0.05. Results were interpreted in terms of *p*‐values, and the effect size was reported where appropriate to indicate the magnitude of the intervention effect.

To determine a sufficient sample size calculation, an analysis was conducted using G*Power based on a previous study (Springer et al. 2013). Assuming a conservative effect size of 0.6, with *α* = 0.05 and power = 0.80, the estimated sample size was 24 participants. To account for a possible 10% drop‐out rate, we increased the final sample to 28 participants (14 per group).

### 2.6 Data Privacy and GDPR Compliance

This study was carried out in strict adherence to the General Data Protection Regulation (GDPR) and other relevant data protection laws. All personal data were collected, processed, and stored in a manner that ensured confidentiality and integrity. To protect participants' privacy, all personally identifiable information was anonymized before any data analysis.

## 3 Results

**FIGURE 1:**
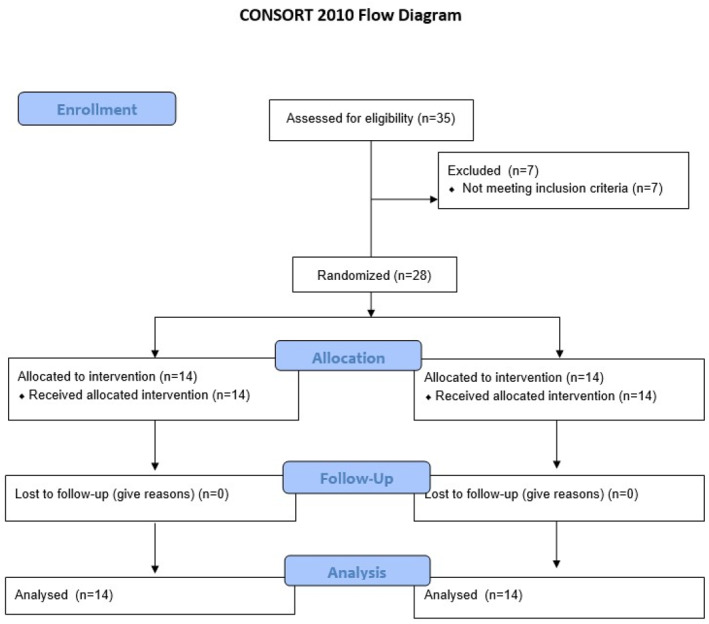
CONSORT flowchart.

Thirty‐five participants were assessed for eligibility. Seven of them were excluded because they did not meet the inclusion criteria due to the presence of concomitant medical conditions or limitations in ankle mobility, as specified in the exclusion criteria. A total of 28 participants (10 female, 18 male, mean age 59.32 ± 12.3) completed the intervention and T1 assessments. Among them, 14 patients were randomized to the FES group, while 14 patients were allocated to the CTR group (Figure 1).

Demographic and clinic characteristics of the participants are reported in Table 1.

**TABLE 1:** Participant characteristics by group.

	FES group	CTR group	*p* value
Participants	14	14	NA
Sex	6F/8M	4F/10M	NA
Left/right hemiparesis	8/6	6/8	NA
Age (years)	57.57 ± 14.25	61.07 ± 10.23	*p* = 0.462
Time since stroke (months)	3.79 ± 2.04 [1–9]	4.78 ± 2.04 [2–9]	*p* = 0.207

*Note:* Data are presented as mean ± standard deviation, or number of participants. Time since stroke is shown as mean ± standard deviation with range in brackets. No significant differences were found between groups for age and time since stroke.

Abbreviations: CTR, control group; F, female; FES, functional electrical stimulation group; NA, not applicable.

**FIGURE 2:**
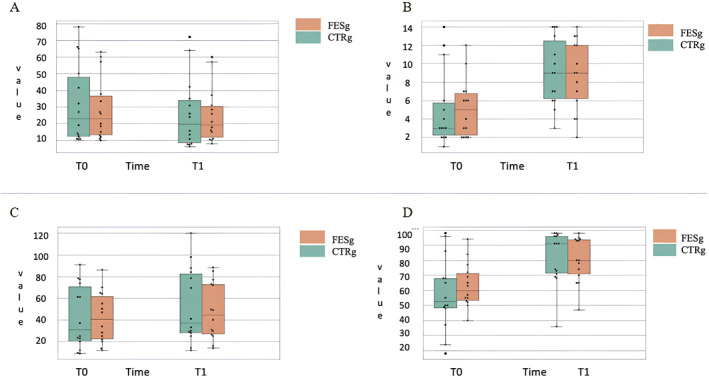
(A) Distribution of RMI scores between T0 and T1. Group 1 (green bar): FES group; Group 2 (orange bar): CTR group. (B) Distribution of 2MWT scores between T0 and T1. (C) Distribution of MBI scores between T0 and T1. (D) Distribution of T25 scores between T0 and T1.

At the baseline, the two groups did not result significantly different for age (FESg: 57.57 ± 14.25 vs. CTRg: 61.07 ± 10.23 years old, *p* = 0.462), and time from the acute event (FESg: 3.78 ± 2.04 vs. CTRg: 4.78 ± 2.04 months, *p* = 0.207). The duration of stroke among participants ranged from 1 to 9 months overall, with 1–9 months in the EXP group and 2–9 months in the CTR group. The results of the Shapiro‐Wilk test indicated that the data for the majority of the outcome measures did not follow a normal distribution (*p* < 0.05), suggesting the need for non‐parametric tests for further analysis. Both the FESg and the CTRg showed significant improvements over time in all outcome measures analyzed, as assessed by the Wilcoxon signed‐rank test. The RMI showed significant improvements in both groups, with *p*‐values of 0.01 for the FES group and 0.01 for the CTR group (Figure 2).

For the 2MWT, significant improvements were observed in both groups, with *p*‐values of 0.01 for the FES group and 0.01 for the CTR group. The MBI, assessing daily living activities, also revealed significant improvements for both groups, with *p*‐values of 0.01 for the FES group and 0.01 for the CTR group. For the T25, both groups demonstrated significant changes, with a *p*‐value of 0.01 for the FES group and 0.05 for the CTR group. The complete results of the Wilcoxon signed‐rank test analysis are presented in the Table 2.

**TABLE 2:** Results of the within‐group analysis.

Test	T0 FES	T1 FES	T0 CTR	T1 CTR	ES FES (*r*)	ES CTR (*r*)
RMI	5.07 ± 4.18	9.14 ± 3.66	5.14 ± 3.16	8.79 ± 3.81	−0.88	−0.88
		(0.01)*		(0.01)*		
MBI	58.00 ± 24.17	82.07 ± 17.80	63.29 ± 14.68	80.43 ± 15.08	−0.88	−0.88
		(0.01)*		(0.01)*		
2MWT	42.98 ± 29.41	53.56 ± 34.93	42.32 ± 23.42	48.29 ± 26.17	−0.88	−0.66
		(0.01)*		(0.01)*		
T25	32.19 ± 23.82	25.94 ± 21.14	28.83 ± 18.86	24.89 ± 16.67	−0.86	−0.53
		(0.01)*		(0.05)*		

*Note:* Complete results of the Wilcoxon signed‐rank test for within‐group comparisons between baseline (T0) and post‐treatment (T1) for both the FES and CTR groups are reported. Values are reported as mean ± standard deviation (*p*‐value).

Abbreviations: 2MWT: two‐minute walk test; ES: effect size; MBI: modified barthel index; RMI: rivermead mobility index; T25: timed 25‐foot walk test.

^*^statistically significant difference (*p* < 0.05) between T0 and T1 within‐group.

The Mann‐Whitney *U* test was performed to compare the differences between FESg and CTRg at both T0 and T1 for all outcome measures. In none of these comparisons were the results statistically significant, indicating that there was no significant difference between the two groups in terms of improvements over time. Additionally, the effect sizes were calculated to assess the magnitude of differences, with values indicating small or negligible effects. The 95% confidence intervals (CIs) were calculated for the between‐group differences in the pre‐post change scores (Δ). The results confirmed the absence of statistically and clinically meaningful differences. Specifically, the CIs for all outcomes included the null value, indicating a high degree of uncertainty around the estimates. Results of Mann‐Whitney *U* test are reported in Table 3.

**TABLE 3:** Between‐group analysis at T0 and T1.

Test Name	T0 comparison (*U*, *p* value)	T1 comparison (*U*, *p* value)	Effect size (*r*)	Mean Δ (FES—CTR) [95% CI]
RMI	88.500, *p* = 0.675	104.000, *p* = 0.800	0.05	+0.43 [–2.06, 2.92]
MBI	77.500, *p* = 0.357	108.500, *p* = 0.645	0.09	+4.24 [–11.24, 19.72]
2MWT	95.500, *p* = 0.927	103.500, *p* = 0.818	0.04	+4.61 [–4.65, 13.86]
T25	102.500, *p* = 0.854	91.000, *p* = 0.765	−0.06	−2.31 [–9.14, 4.52]

*Note:* Mann–Whitney *U* test for between‐group comparisons at baseline (T0) and after the intervention (T1). The test evaluates whether there are significant differences in the distributions of test scores between the two groups. None of the comparisons yielded statistically significant results (all *p*‐values > 0.05), indicating no significant difference between groups at either time point. The effect size (*r*) is also reported to provide a measure of the magnitude of the differences, regardless of statistical significance. The mean difference between groups (FES—CTR) is presented along with the corresponding 95% confidence interval (CI).

Abbreviations: 2MWT: two‐minute walk test; MBI: modified barthel index; RMI: rivermead mobility index; T25: timed 25‐foot walk test.

## 4 Discussion

This study aimed to evaluate the effectiveness of FES as a complementary rehabilitation method in people with stroke. Our findings suggest that integrating FES into conventional rehabilitation enhances overall outcomes, aligning with previous systematic reviews (Zhou et al. 2025; Hwang and Song 2024), who reported that electrical stimulation combined with routine rehabilitation improves motor dysfunction and ADL in stroke patients. Similarly, our results are consistent with those of Everaert and colleagues (Everaert et al. 2013), who demonstrated that FES leads to significant improvements in walking performance over time. However, their study directly compared FES to an ankle‐foot orthosis (AFOs), highlighting how both interventions can be beneficial but act through different mechanisms. This comparison underscores the necessity of individualized rehabilitation approaches, as the effectiveness of each device depends on patient specific factors such as motor function and adaptability. Our findings reinforce this perspective, highlighting that FES should not be seen as a universal solution but rather as a tool to be incorporated into tailored rehabilitation programs. Recently, a study showed (Matsumoto et al. 2023) that FES did not significantly improve the distance covered during the six‐minute walk test. A key confounding factor in studies on FES is the potential use of AFOs in control groups, which may obscure differences in effectiveness. Additionally, commonly used outcome measures useful for assessing walking speed and endurance may lack the sensitivity to detect qualitative improvements in gait.

The lack of superiority of FES over conventional rehabilitation may be attributed to the non‐specificity of the FES protocol used in our study. Our approach primarily focused on optimizing ankle function while neglecting trunk and head stability, which are crucial for improving gait in stroke patients (Sato and Ogawa 2025). This limitation highlights the need for more comprehensive FES protocols that target postural control alongside distal facilitation and work with comprehensive and specific rehabilitation protocols that complement the stimulation technique. Although both groups showed within‐group improvements, between‐group analyses revealed no statistically significant or clinically meaningful differences. The small effect sizes and wide confidence intervals crossing zero indicate high variability and limited evidence for a specific additive benefit of FES. However, variations in stimulation parameters and treatment protocols may contribute to the heterogeneity of findings across studies (Zhou et al. 2025). In our protocol, stimulation was applied at frequencies of 20–50 Hz. Popesco and colleagues (Popesco et al. 2024) suggested 100 Hz as optimal frequency to generate additional force. For this reason, it is possible that the frequency used in our study was not sufficient to maximize central recruitment of motor units. This suboptimal neuromodulation may have limited the potential benefits of FES on functional recovery. Considering our results and the current literature, it is essential to conduct further studies to understand whether the real benefits of FES are determined by electrostimulation or spontaneous patient recovery and which stimulation frequencies are really effective.

Given the critical role of early rehabilitation, further research is needed to differentiate the effects of FES in the subacute and chronic stages of stroke, as these factors may confound group comparisons. The chances of recovery and the influence of rehabilitation have different specific weights according to the distance from the stroke, and this should be considered in forthcoming analyses. Our sample was mainly composed of people in the late subacute stage between 2 and 7 months post stroke. In fact, different from our results in a previous study (Morone et al. 2012), the efficacy of FES has been demonstrated in people with early subacute stage (i.e.< 3 m onset) at both RMI and walking capacity. These different results should be since in the early subacute stage, the proportion of plasticity dependent functional recovery is higher than in the late subacute stage and thus more reactive to an intensive and task oriented training. At the same time, even the foot droop severity might be higher in early moths after stroke and with more possibility to an improvement. Another important factor to consider is the overall severity of the patient's condition, as it may influence responsiveness to treatment. Future studies should investigate this aspect by stratifying patients according to their level of autonomy and motor capacity. However, the current sample size does not allow for such stratification, highlighting the need for larger‐scale trials to explore this variable more effectively.

We assessed motor function using the 2MWT and 25 T because our objective was to evaluate walking speed and capacity. While gait speed and endurance are valuable indicators of functional recovery, they do not fully capture gait stability. To address this limitation, future studies should refine assessment methods by analyzing different gait phases and trunk acceleration symmetry. A key consideration is that while distal external facilitation through FES might intuitively enhance trunk balance, Nevisipour and colleagues (Nevisipour and Honeycutt 2022) demonstrated that FES devices improve users' confidence and safety in initiating a paretic step but do not directly assist with paretic step generation or trunk control. To gain a more comprehensive understanding of gait adaptations induced by FES, future research should incorporate sensor‐based assessments (Tramontano et al. 2024; Bergamini et al. 2017) and optoelectronic instrumentation (Cerfoglio et al. 2024), which offer more precise and reliable biomechanical evaluations.

## 5 Implications on Physiotherapy Practice

FES can be a safe adjunct to conventional gait rehabilitation in stroke survivors. Although no statistically significant superiority was demonstrated over standard therapy, the consistent within‐group improvements highlight the potential of FES to enhance motor recovery, gait speed, and independence in activities of daily living. For physiotherapists, this implies the importance of considering FES as part of a personalized and multimodal rehabilitation approach, especially in patients with foot drop in the subacute stage. Moreover, this study underscores the need for physiotherapists to critically evaluate candidate selection and optimize stimulation parameters to maximize functional gains. The integration of FES should be guided by clinical reasoning, individual patient goals, and the stage of stroke recovery, fostering a more tailored and evidence‐based rehabilitation pathway.

## Author Contributions

**Diego Piatti:** investigation, data curation, writing – original draft preparation. **Giovanni Morone:** writing – original draft preparation. **Antonio Mercuro:** investigation. **Stefano Paolucci:** writing – review and editing. **Marco Tramontano:** conceptualization, methodology, data curation, writing – original draft preparation. **Maria Grazia Grasso:** conceptualization, methodology, data curation, writing – review and editing. All authors have read and agreed to the published version of the manuscript.

## Ethics Statement

The study was conducted in accordance with the Declaration of Helsinki, and was approved by the independent Ethics Committee of Santa Lucia Foundation with the protocol number Prot. CE/PROG.519 28‐09‐15.

## Consent

All the patients involved in this study gave their informed consent.

## Conflicts of Interest

The authors declare no conflicts of interest.

## Data Availability

Data are available upon reasonable request to the corresponding author.
